# Clinical Lecture on Diphtheria

**Published:** 1864-09

**Authors:** John T. Metcalfe

**Affiliations:** Professor Practice of Medicine, University Medical College


### CLINICAL LECTURE ON DIPHTHERIA.

By JOHN T. METCALFE, M.D.,

*Professor Practice of Medicine, University Medical College*

### LECTURE II.

I have said* that, as a rule, the paralysis following diphtheria is prone, by its natural history, to terminate in cure. This we may confirm and expedite by the use of digestible, nutritious food, by the administration of tonics, by friction and kneading of the paralysed parts when practicable, and, occasionally, by the aid of the galvano-electric current. It is well understood that pure air and proper exposure to sunlight are not to be omitted.

I think highly, among the tonic remedies, of the syrup of the phosphates, or what it has been fashionable with some to call chemical food. It is readily taken, agrees well with the stomach, and gives us all the results we should, *à priori*, expect from its composition.

* June number, page 272.

Among tonics of the vegetable class, I prefer strychnia to any other. I do not know that it is better than nux vomica in itself; but the invariable composition of the pure alkaloid or its salts enables us to regulate the dose with perfect accuracy.

I usually write for—Strychniæ, gr. j. Acid. Nitric. Dilut. f. ʒj. Aquæ f. ℥vij. M.

Each minim of this solution contains the one-four-hundred-and-eightieth part of a grain of the salt. To a child of three years I would give from three to five drops in a desertspoonful of water, three times a day.

The beneficial effects from change of air and sea-baths are not less strongly marked in convalescence from diphtherial paralysis than from other adynamic conditions.

The treatment I have thus indicated, gentlemen, is not complicated. It is easy to remember, and I believe embraces nearly everything that is essentially necessary.

As this lecture is intended to anticipate certain questions from you, after graduating, concerning diphtheria, which I would otherwise have put to me by letter, it may not be amiss to say what should be done with regard to your conduct at the breaking out of an epidemic.

You will at once be asked as to *the cause* of the visitation. There is scarcely anything on which the non-professional public insist more strongly, than that a doctor should be able to explain *the reason why* such epidemics prevail. Honesty is the best policy in such cases. Say what is true, "I don't know." Avoid the unwholesome practice of telling people that it is owing to an animal having been drowned in some neighboring pond, from which water is supplied; that it is because the flour or other bread-making material was bad; that the wind blew from some particular quarter; that foolish virgins had put bad oil in their lamps by which the patients read or worked, or the like. We are in ignorance, thus far, of the cause which calls into existence most epidemic diseases. Let us, whilst confessing our darkness, strive and hope for light. As yet it has not been willing to shine on us in such a way as to let us see the reason of diphtherial visitations.

Every one who has had much dealing with diphtheria will recognise the practical wisdom of Dr. William Jenner's division of the disease. It is as follows:

1st. The mild form, in which there is little or no constitutional disturbance, with little or no trouble or pain in swallowing. Albumen is not found in the urine of these cases, which are recognised as diphtherial, by the epidemic prevalence of the disease, and by the patches of exudation revealed by inspection of the fauces or other parts affected.

2d. The inflammatory form. In this, symptoms and signs of more or less severe facial inflammation precede the membranous development; intense injection of the submucous cellular tissue, producing, at times, marked œdema. Febrile action, with great quickness of pulse, is a constant accompaniment of this form.

Doctor Jenner also speaks of the nasal, the laryngeal, the asthenic, and the insidious varieties. They are due simply to certain peculiarities, explicable by their names, and need not detain us longer in their consideration. They will be mentioned again when we come to speak of prognosis. A question which you must be prepared to answer from your patients is, "Doctor, shall I separate the sick child" (for it occurs so much more commonly in children) "from those who are well? Is the disease contagious?"

The usual dissidence among authors as to its contagious nature exists. I advise you very strongly, where it is practicable, to isolate the sick member of the family, and to encourage the removal of the others to some place where the disease does not prevail. If we could be sure that the individual instance was entirely sporadic, it might be proper to give other advice; but, unfortunately, we have too much evidence of its tendency to desolate certain localities, to be able to regard it as otherwise than of epidemic type. You would not hesitate to advise the removal of well people from New Orleans during a visitation of yellow fever, nor from New York whilst cholera prevailed. No more should you hesitate to advise your patients to take their healthy children away from a neighborhood scourged by diphtheria. Setting aside the question of contagion, you are still bound to give the advice indicated. Nor must you forget how the famous French physician Valleix, and our own talented and lamented Dr. Frick, of Baltimore, contracted fatal diphtheria by applying their mouths to the wounds made in tracheotomizing patients threatened with death from suffocation from the laryngeal variety.

The letters I receive from my old friends of former graduating classes almost always indicate anxiety on two points: firstly, what to say in a prognostic way; and, secondly, what it is best to do therapeutically.

Some of those now listening to me have seen with their preceptors many cases of the disease which now occupies us. They have had varied experience. With some the mortality has been very great; with others scarcely any cases have been lost. With nearly all, the greatest number of deaths have occurred at the commencement of the epidemic. This, you know, is observed as a law of these visitations, whether they be of cholera, yellow fever, typhus, dysentery, or what not. Nor is it difficult of explanation. In the earlier days of its prevalence, those most predisposed are those least able to resist the morbific influence. Moreover, this latter seems to grow weaker, to exhaust itself, with time.

As to the other point, why some should be led to think it a disease of little gravity, and others of the most dangerous nature, that is simply owing to the *type of disease* they have encountered. Don't be misled by believing it due to the peculiar excellence of the treatment pursued. I know no better exemplification of what I mean than by referring you to the statistics of enteric fever, as seen in the Massachusetts General Hospital. H re there was the same disease, nosologically speaking; the same treatment prevailed; the hygienic conditions were of the best; the same able men, and of their great ability we can all come up as cheerful witnesses; and yet the mortality indicated was as follows: "In the year 1830, the deaths were one in three and a half; in 1831, they were one in fourteen and a half; and in 1829, one in twenty-five. From 1832 to 1835, inclusive, the mortality was a little less than one in six. From November, 1836, to November, 1838, there were fifty-five successive cases without a single death." Of the prognosis in this, as in other epidemic diseases, speak guardedly in the commencement. No man can know what type the visitation may assume. So also in individual cases, be careful as to what you prognosticate. Some of the most promising cases, in appearance, that I have ever seen, have belonged to the class of insidious diphtheria. There has been little exudation, scarcely any fever, or interference, worth mentioning, with voice or breathing, when suddenly the larynx is invaded and the danger of death is imminent. In general, diphtheria is not fatal provided this last-mentioned complication does not occur. It is possible that asthenia or cardiac paralysis may kill, but in point of fact they very seldom do so. What gives us greatest anxiety is the *uncertainty as to the course* the exudation will take. Should we find it commencing in the posterior and inferior pharyngeal region, extending forwards and upwards, and leaving a healthy mucous membrane behind it, as the exudation is liquefied, we may hope for a happy termination in so far as the most imminent danger is concerned; but, I repeat, until all exudative action has ceased, just anxiety must continue to be felt.

What shall we do in the treatment of a case?

In the first place, attend to the hygienic condition of your patient; see that a comfortable bed be provided; that it be properly placed, with reference to air, light, and noise; and that no more persons be allowed about the sick person than may be necessary to attend to the duties of nursing. Unfortunately, there is a tendency for the female relatives and friends to hold a protracted meeting over children who are ill of diphtheria. I need not tell you how much of the air they breathe and contaminate; how much they talk to and worry the patient; how much they benevolently suggest, each one, her specific remedy, and how much miscellaneous trouble they give. Usually, one person at a time, who has read Miss Nightingale, is all that should be allowed in the sick room.

Having secured this, a cardinal and too much neglected matter, what shall you do in the way of medication?

That will depend on the case you may be called upon to treat. Should it be one of the mild variety, I should advise you to do nothing beyond nursing and giving appropriate nourishment. By this, I mean such nutritious, easily digested food, as we can meet with in the animal broths, milk, and the farinacea. Do not overload the stomach, with the absurd hope that in its somewhat enfeebled state it will be able to go into the digestive business with so much energy as to counteract the natural tendency to debility. Unless for some special reason, the patient should not be made to eat oftener than once in three or four hours, and then the quantity should be carefully observed. I think something might be profitably written on this habit of stuffing sick people, that prevails with some practitioners. I know of no disease in which it is so much the rule as in typhus fever. In this, beef tea and brandy are, by some, systematically given, in the belief that the more the patient can be induced to swallow, the better will be his chance for recovery. Now there is nothing that so certainly tends to provoke restlessness, "nervous irritability," and insomnia, as the presence of material in the stomach which is beyond the digestive power of the patient.

We have a forcible illustration of this in the malaise that occurs from an over distended bladder. I can now recall cases in my experience, where opiates, by the mouth and by injection, valerian, musk, camphor, lavender, and many other antispasmodics, too tedious and useless to mention, had been prescribed by the doctor, without any relief, in which the evacuation of the bladder by catheter, or of the stomach by vomiting, have at once produced the quiet and sleep that know no succedanea as remedial agents.

It is very rarely that a sick person requires so much food as one in health would consume. It may be requisite to give it oftener, but even then we must be careful as to quantity and quality.

When the stomach is rebellious to the point of rejecting whatever is swallowed, I think it safer to resort to external applications in the way of irritants to the epigastrium, and to waiting for tolerance to be established, than to go beyond the simpler means resorted to, such as prussic, carbonic, or the mineral acids, creosote, or a few drops of chloroform. In diphtheria, be very careful of the stomach. Not long ago, I met with a case in which a delicate little girl, suffering from gastric irritability, was made to take a nauseous dose of cinchona infusion every four hours. This was done with a view "to strengthen the child, and to bring about an appetite," notwithstanding that the dose was offensive, and only swallowed after a hard fight, each time.

For some days, strength can be very well supported by the use of nutritious enemata. To these it is wise to resort where sustenance is imperatively called for, and the stomach will not perform its duty. For such enemata, the strong animal broths are preferable. *Let the stomach rest.* In view of the obvious tendency to diminution of the red globules of the blood, as a part of diphtheria, the use of iron would naturally suggest itself. I have, almost always, prescribed small doses of the sesquichloride tincture, administered in cold sweetened water. This is readily taken by children, and is worth much more, in my opinion, than any or than all other so-called constitutional remedies put together. A child three years old will readily take from five to ten drops of the tincture, every two or three hours.

With many practitioners, the chlorate of potash is considered as the chief remedial agent on which dependence should be placed. I have prescribed it largely, but with less satifactory results than seem to have rewarded the experience of some others. Should you employ it, I would recommend you to be careful to avoid the large doses sometimes advised. I have known instances in which severe gastro-intestinal irritation has followed its use.

Where stimulants are indicated, wine-whey, milk-punch, whiskey, or good brandy will be found beneficial. In cases of great thirst, nothing is more grateful nor more easily retained by the patient than mint-julep. Under similar circumstances, you must not forget how useful the carbonic acid water proves; especially where there is irritability of the stomach. Cracked ice is also an excellent remedy.

Our anxious attention, as I have already told you, in this disease, is directed towards the air-passages. Is there any means in our possession by which we can prevent the exudation from reaching them, in the way of topical medication ? I am sorry to say that I do not believe there is. In different published articles on the subject, there are many means advised, such as swabbing the throat with the muriatic tincture of iron, with muriatic acid, with bromine, nitrate of silver, etc.; but I think it safe to advise you not to employ these with the hope indicated. Punching and poking about the tender parts with hard instruments, in my opinion, is much more apt to result in harm than in good.

Some years ago, I thought I had seen benefit from applying the iodide of bromine (four to eight drops to an ounce of gum syrup), by means of a large, soft camel's hair brush. I then believed that it prevented the membrane from extending. In this, I was probably mistaken. The application has one certain good result, whenever there is a sanious discharge from the nose or fœtor of the breath. It acts as an antiseptic. So, no doubt, would creosote or carbonic acid, properly diluted. Nasal injections of liquor of persulphate of iron and glycerine (ʒj to ℥j) are also very serviceable.

In the terrible dyspnœa which accompanies the laryngeal form, the inhalation of steam is more efficacious than any other remedy. The apparatus recommended in Dr. Watson's Practice of Medicine may be resorted to; or the patient may be kept in a room so arranged as to be constantly filled with vapor, at a proper temperature, not less than 98° or 100° F.

In these always bad cases of laryngeal diphtheria, we must try to sustain life until the membrane shall have undergone the liquefactive change to which it has a natural tendency. It will then be expectorated, and we may hope for a cure, provided the exudation has not extended too far down the air-passages.

In conclusion, never fail to keep your patient in bed whilst there is fever or debility. Be careful of premature exposure on account of the impressible state of the general nervous system during convalescence.—*Am. Med. Times.*

